# The Distribution and Composition Characteristics of Siliceous Rocks from Qinzhou Bay-Hangzhou Bay Joint Belt, South China: Constraint on the Tectonic Evolution of Plates in South China

**DOI:** 10.1155/2013/949603

**Published:** 2013-11-03

**Authors:** Hongzhong Li, Mingguo Zhai, Lianchang Zhang, Yongzhang Zhou, Zhijun Yang, Junguo He, Jin Liang, Liuyu Zhou

**Affiliations:** ^1^Key Laboratory of Mineral Resource, Institute of Geology and Geophysics, Chinese Academy of Science, Beijing 100029, China; ^2^Guangdong Provincial Key Lab of Geological Processes and Mineral Resource Survey, Guangzhou 510275, China; ^3^Department of the Earth Science of Sun Yat-sen University, Guangzhou 510275, China

## Abstract

The Qinzhou Bay-Hangzhou Bay joint belt is a significant tectonic zone between the Yangtze and Cathaysian plates, where plentiful hydrothermal siliceous rocks are generated. Here, the authors studied the distribution of the siliceous rocks in the whole tectonic zone, which indicated that the tensional setting was facilitating the development of siliceous rocks of hydrothermal genesis. According to the geochemical characteristics, the Neopalaeozoic siliceous rocks in the north segment of the Qinzhou Bay-Hangzhou Bay joint belt denoted its limited width. In comparison, the Neopalaeozoic Qinzhou Bay-Hangzhou Bay joint belt was diverse for its ocean basin in the different segments and possibly had subduction only in the south segment. The ocean basin of the north and middle segments was limited in its width without subduction and possibly existed as a rift trough that was unable to resist the terrigenous input. In the north segment of the Qinzhou Bay-Hangzhou Bay joint belt, the strata of hydrothermal siliceous rocks in Dongxiang copper-polymetallic ore deposit exhibited alternative cycles with the marine volcanic rocks, volcanic tuff, and metal sulphide. These sedimentary systems were formed in different circumstances, whose alternative cycles indicated the release of internal energy in several cycles gradually from strong to weak.

## 1. Introduction

The Qinzhou Bay-Hangzhou Bay joint belt (QHJB), located between the Yangtze and Cathaysian plates, has attracted much attention because of its interesting tectonic properties. The QHJB is a long, narrow tectonic zone that is shaped like a reversed letter S [1]. It starts from Hangzhou Bay in northeastern Zhejiang province, spanning Zhejiang, Jiangxi, Hunan, Guangdong, and Guangxi provinces, and ends at Qinzhou Bay in the south of Guangxi province. A large amount of research on the QHJB has examined different aspects of its tectonic properties, such as the Caledonian fold belt [2–4], arc-trench system [5–7], melange zone [8], Jiangnan inland orogenic belt [4], South China orogenic belt [9], middle Jiangxi collisional zone [10], South China collisional orogen [11–13], terrane [14], Qinzhou-Hangzhou tectonic joint belt [1], and even the mantle plume structure [15, 16]. Thus far, the orogen properties of the Qinzhou-Hangzhou joint belt [4, 13, 17–20] have been confirmed by the discovery of ophiolite suites [21–23]. These ophiolite suites are distributed in the areas of Fuchan [24–26] and northeast Jiangxi province [27, 28] in the northern segment, areas of northern Guangxi province and western Hunan province [29] in the middle segment, and areas of west Guangdong province [23] and Luchuan [30, 31] in the southern segment. The majority ages of the ophiolite suites lie within the range from 860 Ma to 1030 Ma [31, 32], which indicate, without doubt, Mesoproterozoic and Neoproterozoic orogens for the tectonic properties of the QHJB possibly related to the evolution of the Rodinia supercontinent. The orogen properties of the QHJB remained until the Silurian period [33] and ended with the collisional matching of the Yangtze and Cathaysian plates due to Caledonian movement [34]. 

The Neopalaeozoic tectonic properties of QHJB have puzzled researchers, and major controversies exist in regard to the existence of an ocean basin with subduction. In published papers, the Neopalaeozoic ophiolitic melange in northeast Jiangxi province possibly indicates the Qinzhou-Hangzhou tectonic zone to be the eastern part of the Neopalaeozoic Tethys ocean [28], and the early Mesozoic acid volcanic rocks of the south segment in southwest Guangxi province confirm the subduction of an ocean basin until the middle Triassic period [35]. Although there is diversity among the north, middle, and south segments [36–38], the QHJB exhibits integral uniformity in its tectonic evolution [36, 39]. Therefore, the tectonic properties of the QHJB were proposed to be an orogen in the Neopalaeozoic by the current authors [28, 35]. The Qin-Fang trough has been attested in the south segment [40, 41], but there is a lack of orogenic properties because of the absence of a homochronous ophiolite suite and volcanic rocks in the middle and north segments [31]. This indicates that the Neopalaeozoic tectonic properties for the entire QHJB remain somewhat unclear, and the orogenic properties may not possibly run throughout the whole QHJB with subduction in the ocean basin. In addition, the QHJB possibly underwent rifting in the NE direction in the Devonian and early Permian and then experienced folding and orogeny due to the compression of Dongwu movement [33]. The tectonic properties of the QHJB remain unclear in the Hercynian.

 The distribution, composition, and microfabric characteristics are significant for understanding the geological properties of the QHJB. In the literature, the geologic setting and geographic framework can be proposed using siliceous rocks for its geochemical characteristics [42–44], which helps underpin the tectonic framework with siliceous rocks widely [45–47]. In the QHJB, the siliceous rocks are distributed widely [38] and indicate hydrothermal genesis [28, 40, 48–50] and can possibly contribute to understanding the geological properties of the QHJB. Although these previous studies examined some individual cases, rarely has attention been given to the temporal and spatial distribution of siliceous rocks in the entirety of the QHJB. In the QHJB, the siliceous rocks have complex tectonic evolution [38], and they potentially preserve significant evidence that will aid in the comprehension and understanding of tectonic properties. Thus, the distributions of siliceous rocks in the entirety of the QHJB were examined in this study to determine the tectonic significance of the siliceous rocks. In addition, geological analysis, X-ray diffraction (XRD), scanning electron microscopy (SEM), and energy dispersive spectrometry (EDS) were also adopted for analysis of the Neopalaeozoic siliceous rocks. These analyses aided in understanding the geological characteristics and associated indications. 

## 2. Geological Setting and Petrological Characteristics

### 2.1. Geological Setting

**Figure 1:**
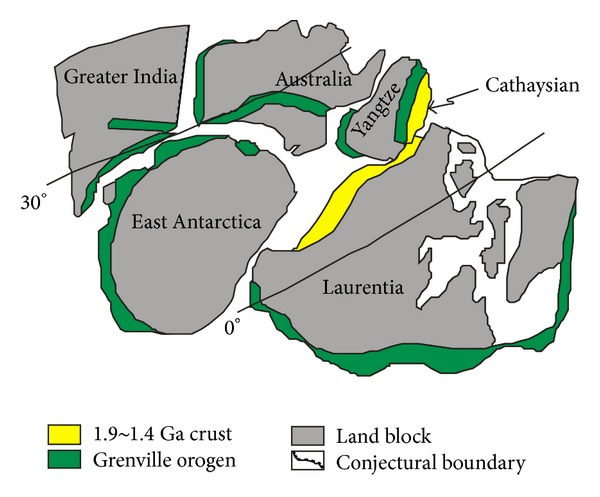
Location of South China block in north late Mesoproterozoic Rodinia supercontinent (after [54]).

The Mesoproterozoic South China block (also named as Elder South China block) is distributed among three land blocks (Figure 1), which are named East-Australia, Antarctica, and Laurentia, respectively. The Elder South China block is originated from the matching of Yangtze and Cathaysian blocks, whose crystalline basement is formed in diverse geological era. In the previous study, the ancient land crusts of Yangtze and Cathaysian are, respectively, formed in the Neoarchean and Paleoproterozoic [51]. The formation of the Yangtze ancient land crust is attested to be in Neoarchean as evidenced by the crystalline basement of the Neoarchean to Paleoproterozoic [52]. The Cathaysian ancient land crust is a group of similar crusts including Yunkai, Mintai, and Wuyi platforms and is formed at the end of the Paleoproterozoic (1800 Ma) for the Paleoproterozoic crystalline basement [51]. These two blocks are cohered together possibly due to the Lüliang movement, which is witnessed by the Lüliangian granite in southwest Zhejiang province [34]. With the matching of Yangtze and Cathaysian blocks, the Elder South China block [53] is formed at the end of late Paleoproterozoic [33]. Then, the whole Elder South China block was covered by a uniform sedimentary veneer.

**Figure 2:**
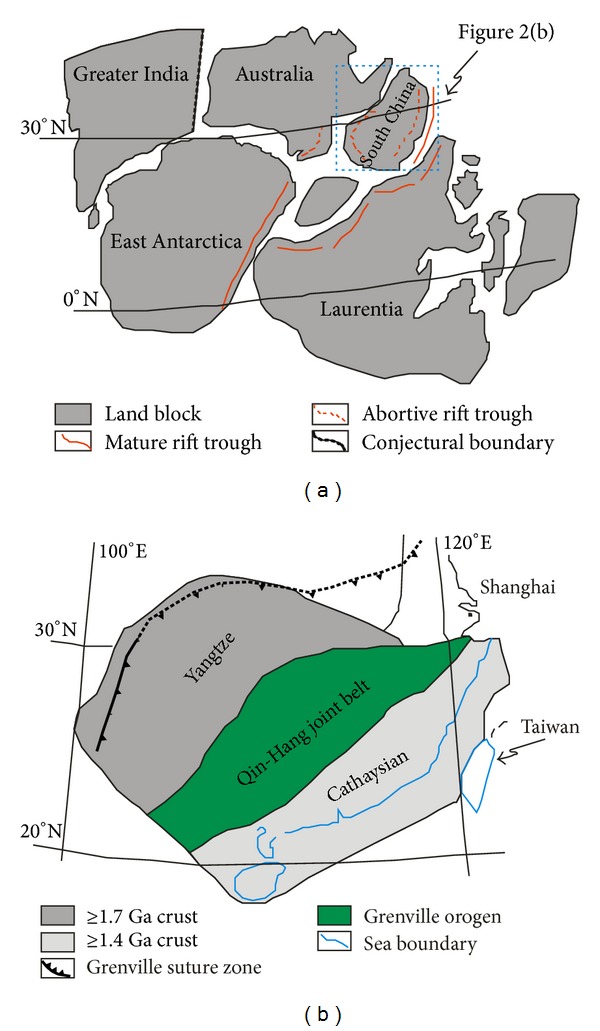
Early Neoproterozoic configuration of north Rodinia supercontinent (a) and South China block (b) ((a) after [54]; (b) after [57]).

The QHJB comes from the fragmentation of the Elder South China block at the beginning of the Mesoproterozoic. There is a fragmentation period after Lüliang movement with rift troughs, and this fragmentation contributes to the primary ocean basin of QHJB which separates the Yangtze block from Cathaysian block. After its formation, the QHJB undergoes cyclic tectonic movements, which is possibly associated with the evolution of the Rodinia supercontinent. During the whole tectonic evolution process, there are three tectonic cycles from Changchengian period to Silurian for the QHJB [33]: the first tectonic cycle ends up with the first episode of Jinning movement (~1000 Ma), which is from the Changchengian period to the Jixianian period; the second tectonic cycle ends up with the second episode of Jinning movement (~850 Ma), which is just during the Qingbaikou period; the third tectonic cycle ends up with the Caledonian movement, which is from the Sinian period to the Silurian period. At the end of the second tectonic cycle, when the Elder South China ocean is closed with collisional matching, the Yangtze and Cathaysian blocks are cohered together to form South China block which is a part of Rodinia supercontinent [55]. Affected by the fragmentation events of Rodinia supercontinent (Figure 2(a)), the South China block is splitted up into Yangtze and Cathaysian blocks again (Figure 2(b)). Additionally, the prevenient Cathaysian block is further splitted up into three slight blocks with rift troughs as insulation, and these small blocks are named South Zhejiang—North Fujian mountain, Middle Jiangxi—South Jiangxi mountain and Yunkai mountain [31]. There is a collisional matching for the Yangtze and Cathaysian plates during the Caledonian event, and the two plates are cohered to a unified South China block again which agrees with the formation of Gondwana supercontinent. As a part of the Gondwana supercontinent, the South China block is covered by the Neodevonian uniform sedimentary veneer entirely. There are several cycles of tension and compression after the Caledonian movement [34], when the whole South China plate is resplitted up again with the separation of Yangtze and Cathaysian plates. Between Yangtze and Cathaysian plates, there also exists the QHJB, whose tectonic property remains an uncertainty and will be discussed in this paper. In the published papers, the QHJB is an ocean basin due to the Neopaleozoic ophiolitic melange [28] and early Mesozoic acid volcanic rocks [35], but it is also treated to be an inland fault zone and rift [56] for the absence of ophiolitic and homochronous magmatic rocks since Sinian [31]. During the Hercynian and Indosinian, the tension ends up with compression contributed by the final Hercynian movement and Indosinian movement. The ultimate matching for the Yangtze and Cathaysian plates is due to the Dongwuian event in Permian [41], and the whole plate solidified as the result of the Yanshan movement [56].

### 2.2. Geological Characteristics of Dongxiang Area

**Figure 3:**
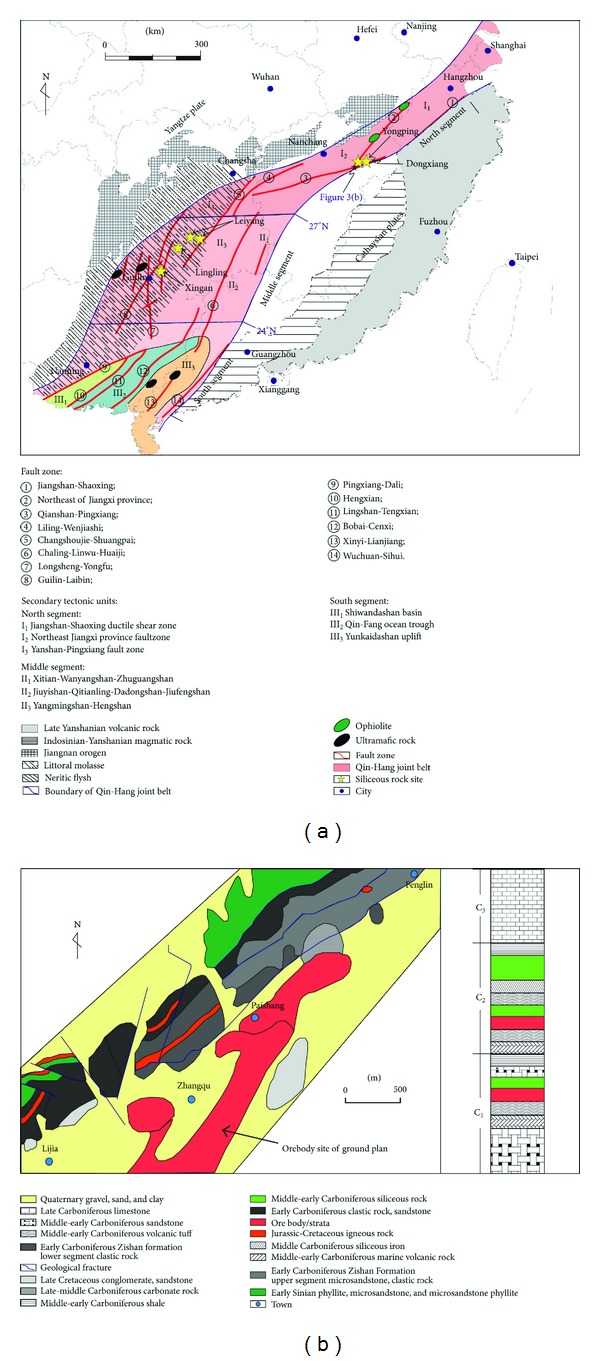
Geological map of QHJB (a) and Dongxiang area (b) ((a) after [36]; (b) after [48, 58]).

The QHJB is divided into north, middle, and south segments due to the diversities in metallogenesis and geological evolution. In the previous studies, there are obvious differences in QHJB for its metallogenesis [37, 39], hydrothermal sedimentation [38], and geological evolution [36], which denote a subsection with latitude lines of 24°N and 27°N (Figure 3(a)). According to this, the QHJB is divided into north, middle, and south segments, and the middle segment of QHJB (24–27°N) is in accordance with Nanling mountain chain. In north segment, there is a Dongxiang copper-polymetallic ore deposit which is located in the Xinjiang basin at the south margin of the Jiangnan terrane. The Xinjiang basin is a secondary fault basin, which is distributed across the west of the Qiantangjiang-Xinjiang taphrogenic trough [48, 49]. In the north segment of QHJB, there are two deep faults running in ENE direction, which are Qianshan-Pingxiang fault and Northeast Jiangxi-Qianshan-Pingxiang fault. In addition, the Dongxiang ore district is distributed in the northeast of Northeast Jiangxi fault and is located in the northwest of Qianshan-Pingxiang fault. 

The regional geology is simple relatively in the Dongxiang ore district. There are strata in three periods in the Dongxiang ore deposit, Fuzhou city, Jiangxi province, and South China (Figure 3(b)). The rocks in the late Cretaceous strata include purple sandstone and conglomerate. The Carboniferous strata are mainly composed of clastic rock with interlayered volcanic clastic. In the Proterozoic strata, there are pelitic, limestone, and microsandstones. The metamorphism is relatively slight, and these rocks only partially metamorphosed into phyllite. The magmatism is weak with dykes of granite porphyry, granodiorite porphyry, and rhyolite. The strata generally form a monocline, which changes slightly with an approximate occurrence of 145°*∠*35°. Numerous fractures are distributed in the Dongxiang mining area, and their outcrops are on strikes of NE-ENE, SN, and NW directions. Additionally, the fractures along the NE-ENE strike represent a stripping fault, which are manifested as the main ore controlling fractures.

The Neopaleozoic siliceous rocks are collected from the mining area of Dongxiang area in the north segment of Qinzhou-Hangzhou joint belt. In the Xinjiang basin, there are several VMS-type polymetallic ore deposits including Dongxiang ore deposit, Yongping ore deposit, and Lehua ore deposit, and they exhibit intergrowth with siliceous rocks (Figure 3(b)). The siliceous rocks have outcropping conformably to the strata, which are either sill-like or conformable. The strata of siliceous rocks, with a thickness of up to 20 meters, are located in Carboniferous strata, which are located below the land surface about 100 meters to 150 meters. These siliceous rocks are often accompanied by carboniferous marine volcanic rock systems and massive sulphide-rich ore strata. In this paper, the siliceous rocks were collected from the mining area of Dongxiang copper-polymetallic ore deposit, Fuzhou city, Jiangxi province, South China, which is located in the north segment of Qinzhou-Hangzhou joint belt.

### 2.3. Petrological Characteristics

**Figure 4:**
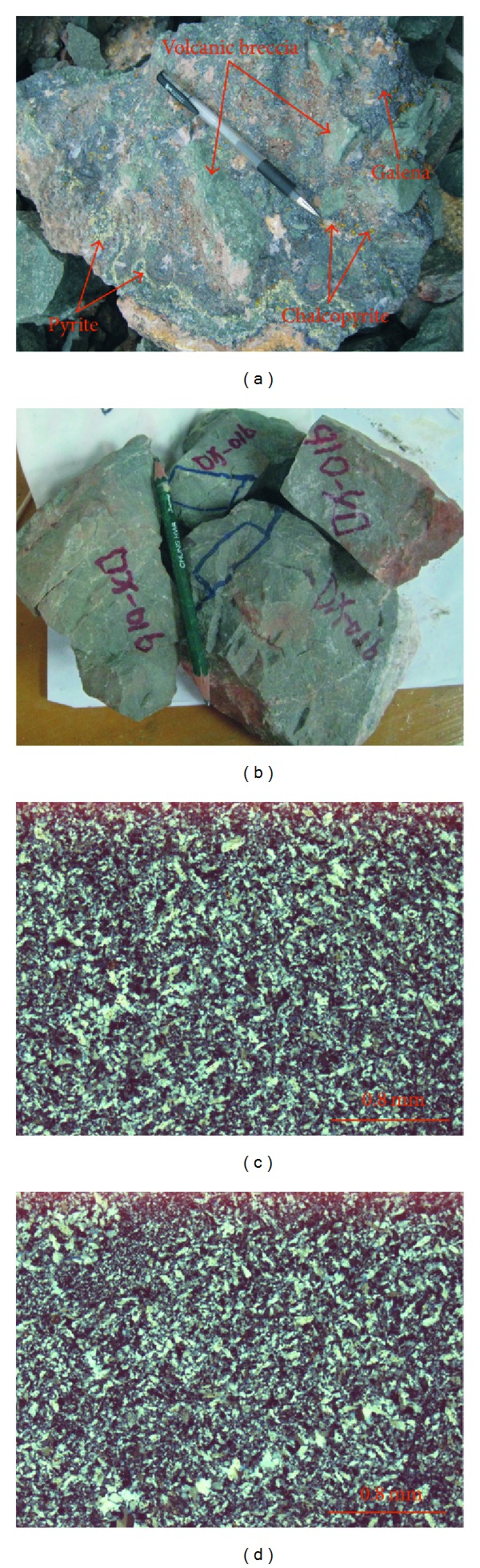
Siliceous rock from the Dongxiang ore deposits region ((a) ore including pyrite, galena, and chalcopyrite; (b) grey samples of siliceous rock; (c) fine-grained siliceous rock; (d) quartz-veined siliceous rock).

In the Dongxiang copper mining area, the sulfide ore (Figure 4(a)) and siliceous rocks (Figure 4(b)) were collected from the mine below the land surface. The ore were composed of volcanic breccia and metal sulphide minerals (Figure 4(a)), and the volcanic breccias were cemented by the metal sulphide minerals (e.g., pyrite, chalcopyrite, galena, and sphalerite). The galena and sphalerite were shown to be coarse-grained breccia (Figure 4(a)), while the minor chalcopyrites were scattered and coarse-grained. Additionally, the pyrites in small quantity were distributed in the fissures among volcanic breccias with vein shape. The siliceous rocks were grey and compact (Figure 4(b)) and consisted of authigenic quartz grains with low crystallinity microscopically (Figure 4(c)). The fine-grained or micrograined quartz grains were shown to be less automorphic and have closely-packed texture, which is in high agreement with the siliceous rocks originated from hydrothermal sedimentation [45, 59]. Furthermore, slight recrystallization happened to the siliceous rocks with coarser grains, which were sometimes arranged in a line like a quartz vein (Figure 4(d)).

## 3. Samples and Experiments

In the pretreatment processes, fresh samples were selected, cleaned in ultrapure water, dried, and then divided into two groups. One group was polished into thin sections (≤0.03 mm), and the other group was broken into grains of 3 mm diameter, using cleaned corundum jaw breakers. Some of that sample was selected, cleaned, redried, and ground into grains with diameter of 0.075 mm in an agate ball mill (NO. XQN-500x4). Additionally, all the ore-hosted siliceous rocks were separated from the ore several times to purify them.

The pretreatment and analysis of the principal elements were carried out in the State Key Laboratory of Ore Deposit Geochemistry, Institute of Geochemistry, Chinese Academy of Sciences. In this study, the SiO_2_ content was analysed by classic gravimetric method. The Al_2_O_3_ content was analysed by EDTA titrimetric method (content > 1%, accuracy of 1.5%). The TiO_2_ and P_2_O_5_ contents were analysed by colorimetric method. The K_2_O, Na_2_O, MnO, CaO, and MgO contents were analysed by atomic absorption spectroscopy (instrument type PE-5100), to an accuracy of 0.2%. The FeO and Fe_2_O_3_ contents were obtained by potassium dichromate titration method.

Inductively coupled plasma mass spectrometry (ICP-MS, instrument model: PE Elan 6000) was carried out in the State Key Laboratory of Geological Processes and Mineral Resources, China University of Geosciences. The analysis accuracy of the ICP-MS lay between 1% and 3% and was used to test the presence of trace and rare earth elements. The test was prepared from an acid-soluble solution in accordance with the standard process. Samples were weighed out to 100 mg and then placed in the sealed Teflon cell before the addition of l mL concentrated HF and 0.3 mL 1 : 1 HNO_3_. After ultrasonic oscillation, samples were placed on a 150°C hot plate and then evaporated to dryness, rejoined with the same amount of HF and HNO_3_, and heated under confinement for a week (at about 100°C). After evaporation and dissolution by 2 mL of 1 : 1 nitric acid, the sample was diluted 2000-fold and finally tested by PE Elan 6000 ICP-MS. The results of trace element concentrations are shown in Tables 2 and 3, respectively.

The pre-treatment for the XRD analysis was performed in the Guangdong Provincial Key Laboratory of Geological Processes and Mineral Resource Survey. XRD analysis was carried out in the laboratory of the College of Chemistry and Chemical Engineering of Sun Yat-sen University. XRD data were collected with an X-ray powder diffractometer (instrument type: D/Max-2200 vpc) using the reflection focusing geometry (Cu K*α* radiation; 40 kV at 30 mA; scanning speed: 0.12 s per step; step length: 0.02°; continuous scanning mode). Over the range of scanning angle from 5° to 100°, the data were analysed by JADE-5.0 software with the eight highest peaks as the basis for their identification of each mineral type.

The EPMA analysis was carried out by the State Key Laboratory of Ore Deposit Geochemistry, Institute of Geochemistry, Chinese Academy of Sciences. The instrument type was a JEOL JSM-6460LV (20 kV), and the X-ray EDS was produced by the EDAX company. The resolution of the instrument was 3.0 nm and the magnification is 5 ~ 300,000.

## 4. Characteristics of Distribution and Geochemistry

### 4.1. Distribution Characteristics

In South China (Figure 3(a)), the QHJB was 2000 kilometres in length and 100~150 kilometres in width [31]. In this study, the distribution of siliceous rocks was calculated with the basic unit of county. When the siliceous rocks were distributed in the uniform place with diverse strata, these siliceous rocks would be separated with basic unit of formation. So, the siliceous rocks were calculated with formation and county as the temporal unit and spatial unit, respectively, whose distribution category included Zhejiang, Jiangxi, Hunan, Guangdong, and Guangxi provinces. What is more, Precambrian strata were divided into Mesoproterozoic and Neoproterozoic (Sinian) strata cursorily according to the literature [60–64]. Calculated from the regional geology of Zhejiang, Jiangxi, Hunan, Guangdong, and Guangxi provinces [60–64], the temporal and spatial distributions of siliceous rocks in QHJB were listed in Table 1.

#### 4.1.1. Temporal Distribution

**Figure 5:**
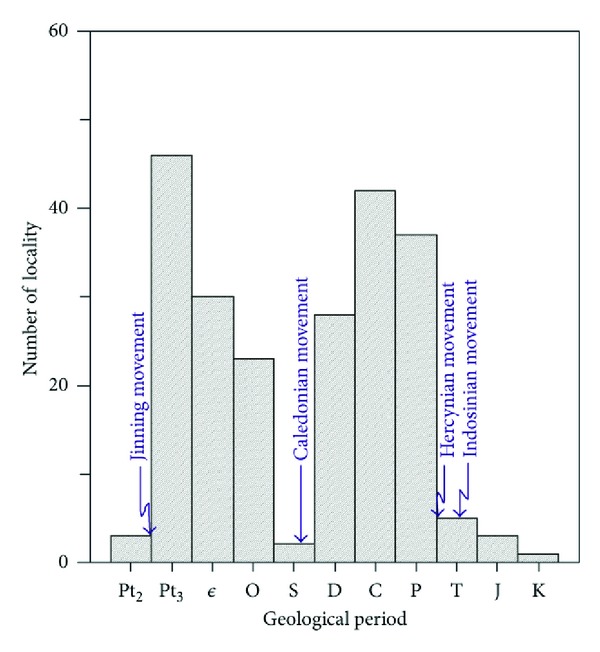
Column diagram of localities for siliceous rocks in QHJB (data were calculated from the literature [60–64]).

The siliceous rocks were easy to develop more widely in tensional setting and decreased due to the compression setting. In QHJB (Table 1), the marine siliceous rocks were distributed from Mesoproterozoic to Cretaceous, whose widest eras of distribution of siliceous rocks were Neoproterozoic, Carboniferous, and Permian. The Neoproterozoic siliceous rocks were in the largest scale, which was possibly contributed by the long geological history. There were two phases with siliceous rocks in larger scale (Figure 5), which were in Caledonian and Hercynian, respectively: the first period for siliceous rocks in large scale was from Neoproterozoic to Ordovician and ended up with scale decreasing abruptly due to the Caledonian movement in Silurian; the second period for siliceous rocks in large scale was from Devonian to Permian and ended up with a sudden reduce in scale due to the Hercynian movement in the end of Permian. Since Triassic, the scales of siliceous rocks persisted to diminish, which were possibly contributed by the regression of the whole of South China. In QHJB, the geological setting was tensional in the Caledonian and Hercynian [31, 33], when the siliceous rocks had large scale with the widest distribution due to these geological settings (Figure 5). However, the Caledonian movement and Hercynian movement contributed to compressional setting [34, 41], when the siliceous rocks showed small scale with the widest distribution due to the compressional setting. According to this, the widest distributions of siliceous rocks agreed with the tensional setting, whereas the decreasing distributions of siliceous rocks contributed by the compressional setting due to the geological movement. So, the siliceous rocks were preferential to develop more widely in tensional setting in the QHJB and decreased due to the compressional setting.

#### 4.1.2. Spatial Distribution

**Figure 6:**
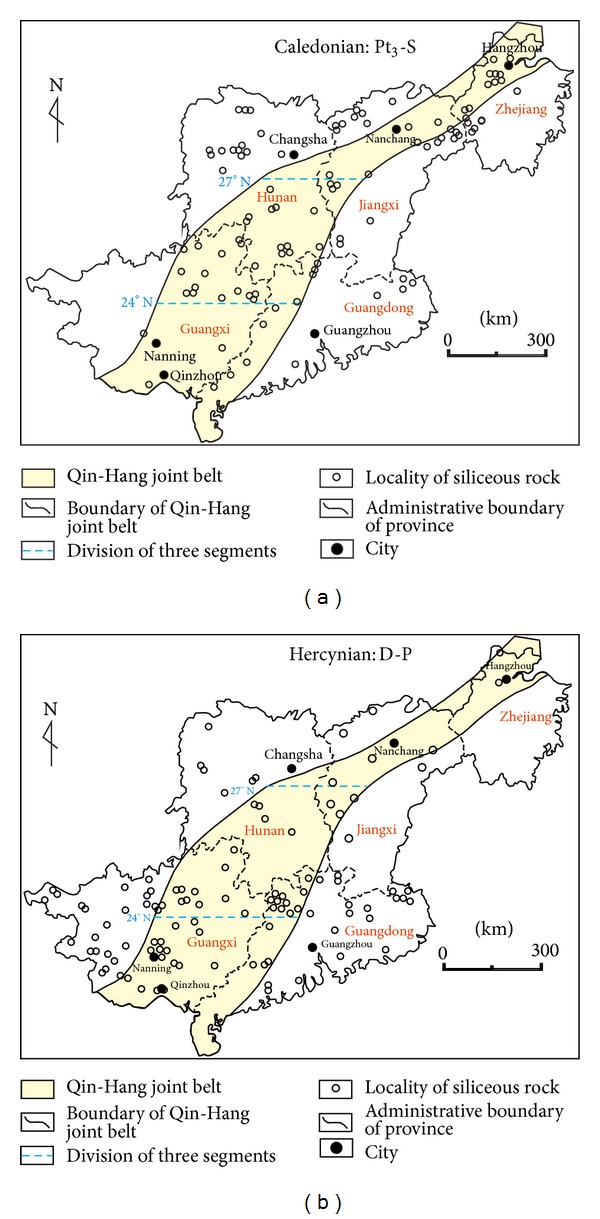
Distribution of siliceous rocks in large scale in Southeast China ((a) Caledonian period; (b) Hercynian period).

The distribution of siliceous rocks acted as indication of the geological setting, and the tensional setting was propitious for the development of siliceous rocks. The siliceous rocks were widely distributed in Southeast China, whose majority were located in the category of inner and adjacency QHJB (Figures 6(a) and 6(b)). In the previous study, the QHJB was divided into north, middle (24–27°), and south segments [36]. According to this, the Caledonian siliceous rocks had majority in the north and middle segments, whereas the major siliceous rocks were located in middle and south segments in Hercynian. In the published paper, the breakup of South China started from the north segment possibly due to the mantle plume with transfer in southwest direction [55], and the collisional matching also began from north segment extending in southwest direction [33]. Based on the aforementioned, a tensional setting agreed with the siliceous rocks developing in a larger scale relatively, whereas the compressional setting was adverse to the development of siliceous rocks. According to this, the diversities in spatial distribution of siliceous rocks came from the hysteresis on geological setting, which came from the passing of geological setting from north segment to south segment. In the Caledonian, the tensional setting was too late to the evolution of siliceous rocks in the south segment, which meant a relatively shorter time for the sedimentation of siliceous rocks in the south segment than north and middle segments. In the Hercynian, the compressional setting was too early to prevent the evolution of siliceous rocks, which contributed to a long time for the development of siliceous rocks in the south segment than north and middle segments. In addition, the distribution of siliceous rocks acted as indication of the geological setting, which meant the tensional setting had favorableness to the development of siliceous rocks.

### 4.2. Geochemical Characteristics

There were geochemical indicators of major and trace elements for siliceous rocks to identify their genesis, sedimentary environments, and geological setting. In the north segment of QHJB, the geochemical composition of the siliceous rocks in Dongxiang ore area was listed in Tables 2–4, which denoted the genesis and sedimentary environments as follows.

**Figure 7:**
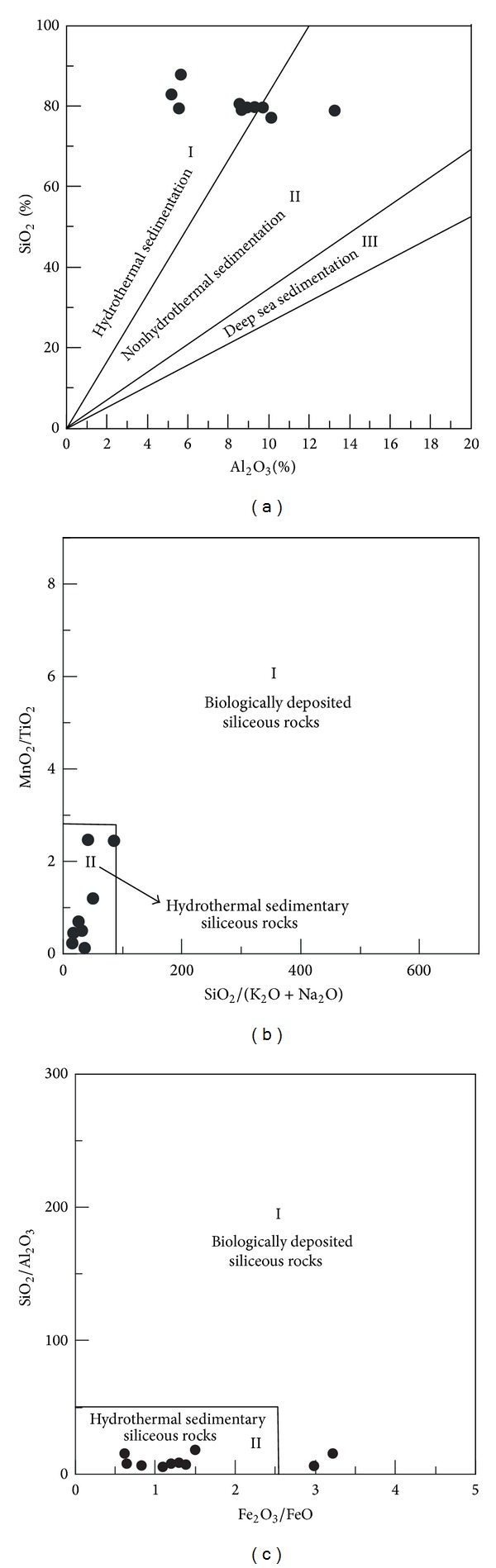
Major element discrimination diagrams for siliceous rocks of Dongxiang area ((a) after [72]; (b) after [73]).

**Figure 8:**
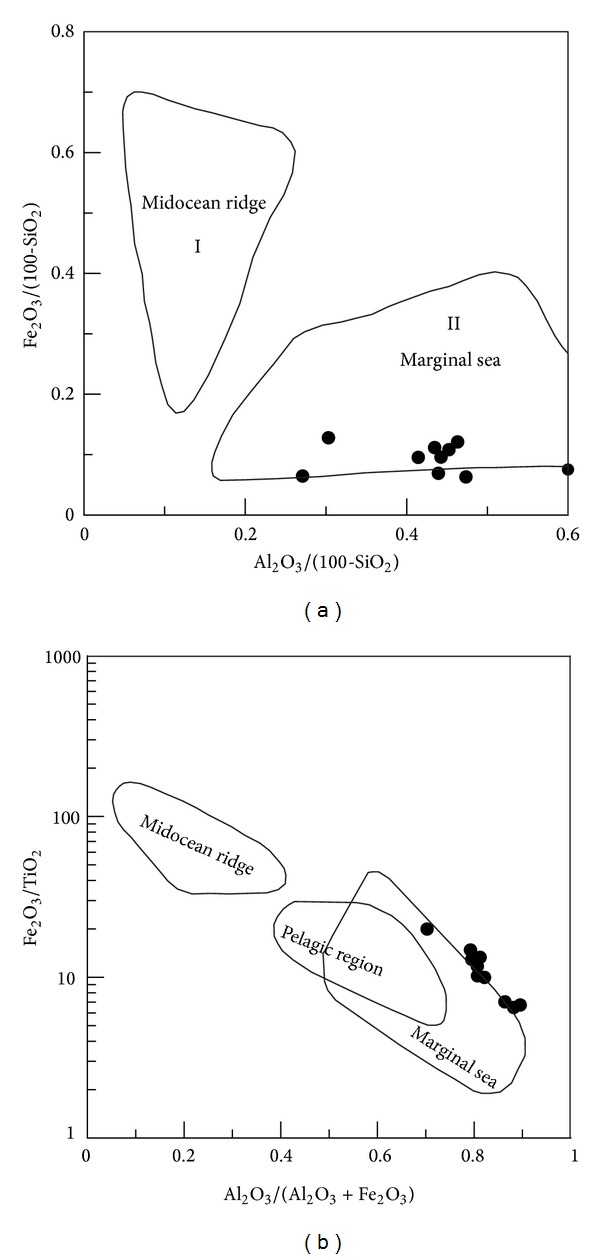
Major element discrimination diagrams for siliceous rocks of Dongxiang area (after [73]).

**Figure 9:**
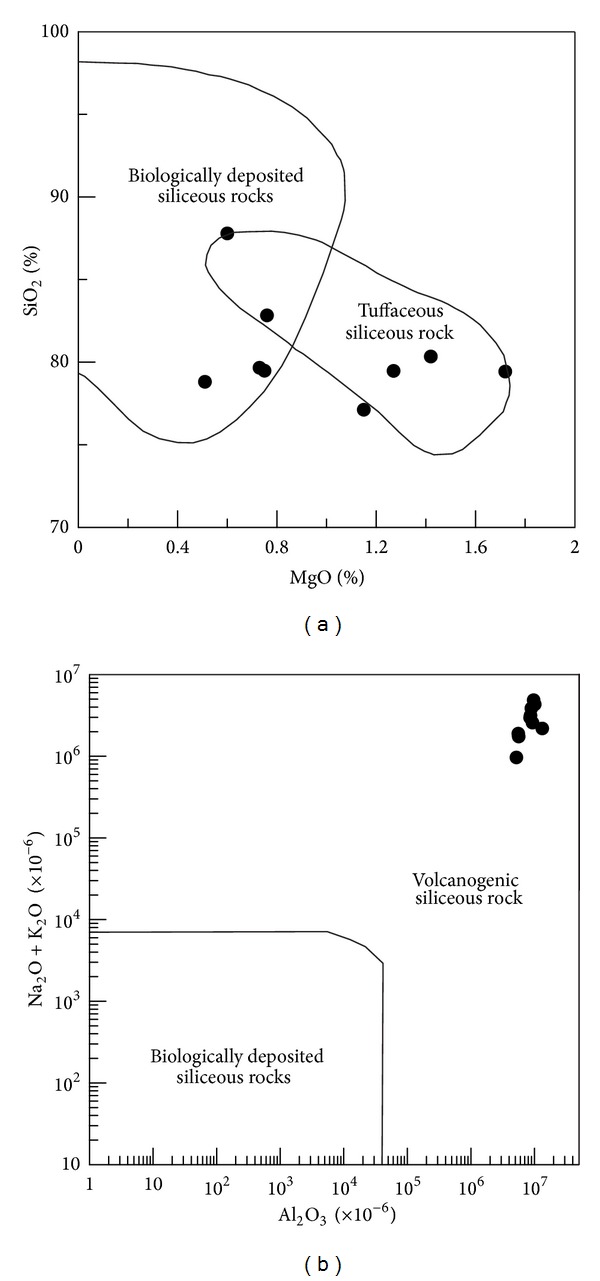
Major element discrimination diagram of siliceous rocks of Dongxiang area (after [73]).

**Figure 10:**
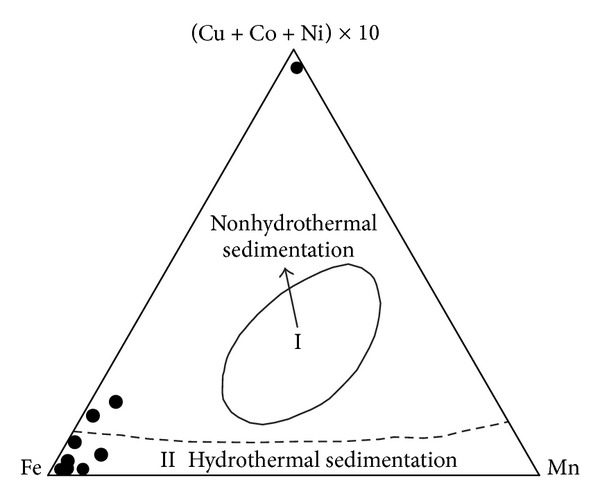
Major element discrimination diagram of siliceous rocks in Dongxiang area (after [81]).

**Figure 11:**
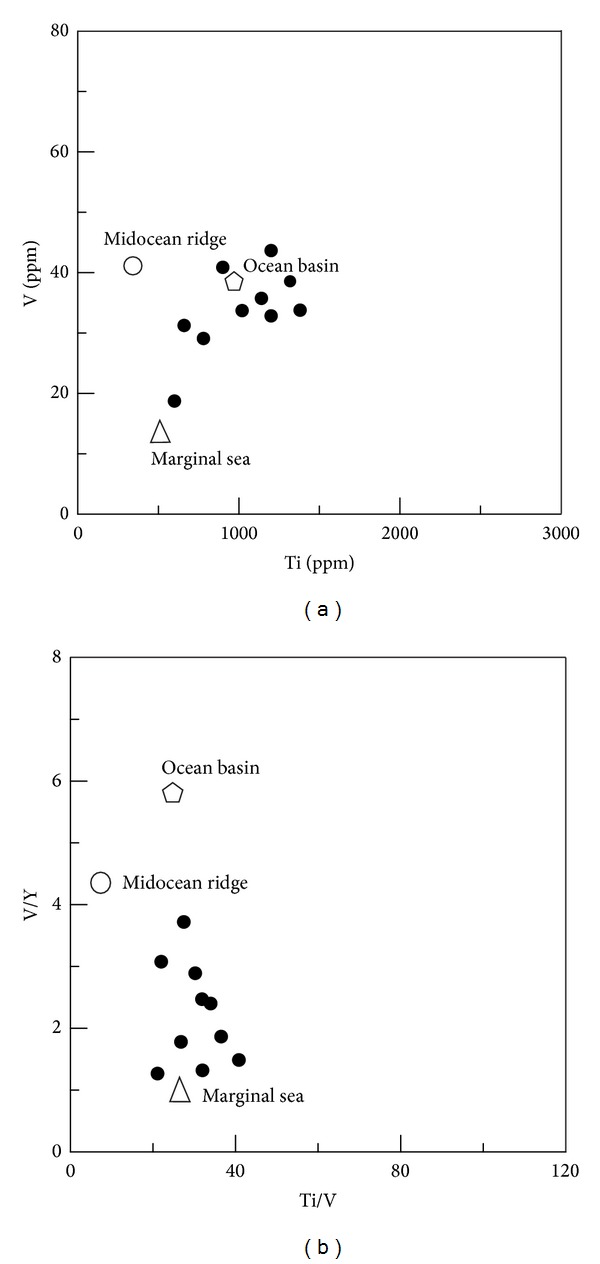
Trace element discrimination diagram by formation environment for siliceous rocks from Dongxiang (after [84]).

**Figure 12:**
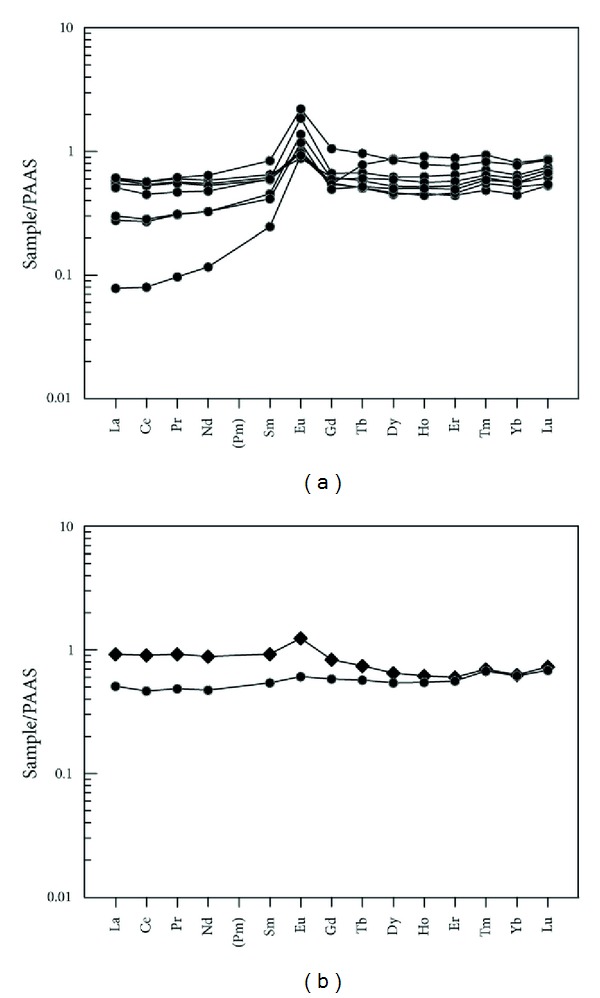
PAAS normalized REE patterns for siliceous rocks from Dongxiang.

**Figure 13:**
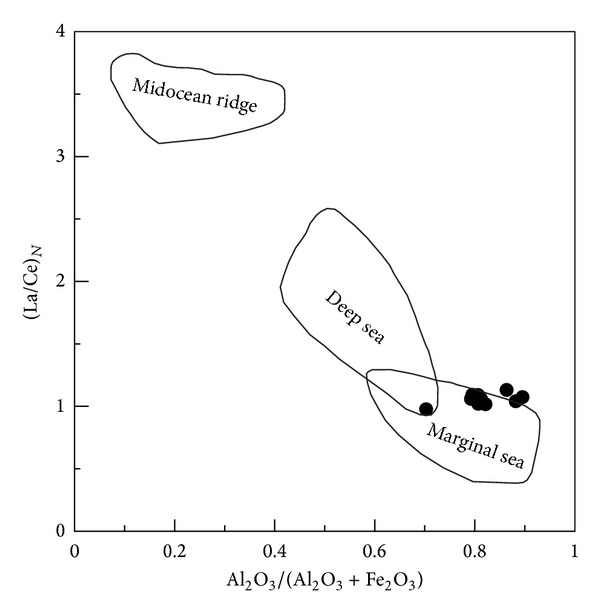
Major elements and REE discrimination diagram by formation environment for siliceous rocks from Dongxiang (after [73]).

(1) The major elements of the siliceous rock from Dongxiang ore area were shown in Table 2, and they indicated the following information.

(a) The siliceous rocks were originated from hydrothermal sedimentation. The siliceous rocks had SiO_2_ ranged from 77.13% to 87.80% with an average of 80.41%. The Si/Al ratios lay between 5.24 and 14.04 with average of 9.13, which were lower than those of pure siliceous rock (80 to 1400) [67]. The Al/(Al + Fe + Mn) values lay between 0.48 and 0.82 (average = 0.63), which were higher than those of typical hydrothermal siliceous rocks with Al/(Fe + Al + Mn) < 0.4 [68]. The MgO content ranged from 0.60% to 2.50% (average = 1.14%), which were higher than those of pure hydrothermal siliceous rocks [69]. The Fe/Ti ratios ranged from 10.79 to 41.95 (average = 25.70) and were basically consistent with those of typical hydrothermal sedimentary siliceous rocks with Fe/Ti > 20 [70]. The (Fe + Mn)/Ti values ranged from 10.83 to 45.12 (average = 26.89), which were consistent with those of typical hydrothermal siliceous rock with (Fe + Mn)/Ti > 20 ± 5 [70]. The Fe_2_O_3_/FeO ratios ranged from 0.63 to 3.22 (average = 1.45), which were similar to those of typical hydrothermal siliceous rock being 0.51 [71]. As a whole, the geochemical characteristics above generally matched those of hydrothermal siliceous rocks and corresponded to the associated geochemical discrimination diagrams (Figures 7(a), 7(b), and 7(c)). Additionally, some of the geochemical indicators, such as the Al/(Al + Fe + Mn) values and the MgO contents, deviated slightly from those values exhibited by classic hydrothermal material, which possibly indicated the influence of terrigenous input or biological activities (Figure 7(c)). So, the siliceous rocks were originated from hydrothermal sedimentation and were possibly influenced by terrigenous input or biological activities.

(b) The siliceous rocks deposited in a basin of the marginal sea. The Al/(Al + Fe + Mn) values ranged from 0.48 to 0.82 (average = 0.63), which indicated the siliceous rocks of the marginal sea [42]. The MnO/TiO_2_ ratios ranged from 0.03 to 2.46 (average = 0.92), which were approximately consistent with those of siliceous rocks formed in the marginal sea [74]. The Al/(Al + Fe) values lay between 0.50 and 0.82 (average = 0.64), which were consistent with those of bedded siliceous rocks in the marginal seas [69]. The Al_2_O_3_/(Al_2_O_3_ + Fe_2_O_3_) values ranged from 0.70 to 0.90 (average = 0.82), which were consistent with those of typical siliceous rock with Al_2_O_3_/(Al_2_O_3_ + Fe_2_O_3_) > 0.7 from the continental margins [73]. So, these geochemical characteristics denoted the siliceous rocks deposited in the marginal sea, which was strongly supported by the associated geochemical discrimination diagrams (Figures 8(a) and 8(b)).

(c) The siliceous rocks had close relationship with volcanic activity. The K_2_O/Na_2_O ratios ranged from 0.23 to 2.53 (average = 1.46), and the samples with K_2_O/Na_2_O < 1 agreed with those of the typical siliceous rocks related to submarine volcanism [69]. The SiO_2_/(K_2_O + Na_2_O) values ranged from 16.26 to 85.40 (average = 35.05), which were approximately consistent with those of siliceous rock arising from chemical sedimentation related to volcanic eruptions [75]. The SiO_2_/Al_2_O_3_ ratios ranged from 5.94 to 15.93 (average = 10.35), which approximately corresponded to those of siliceous rock originated from magmatism with SiO_2_/Al_2_O_3_ < 13.7 [76]. The SiO_2_/MgO ratios ranged from 31.64 to 154.55 (average = 88.91), which were slightly higher than that of typical siliceous rocks related to magmatism with SiO_2_/MgO < 69.5 [76]. The Al_2_O_3_/TiO_2_ ratios ranged from 42.77 to 57.73 (average = 50.03), which were consistent with those of siliceous rock with source area of magmatic rock [44]. In conclusion, the siliceous rock was shown to have genesis related to magmatism, which was strongly supported by the geochemical discrimination diagrams (Figures 9(a) and 9(b)). Additionally, there were slight biological contributions in the siliceous rocks (Figure 9(a)). So, the sedimentation for the siliceous rocks was affected by the volcanic eruption.

 (2) The trace elements of the siliceous rock from Dongxiang ore area were presented in Table 3, and they mainly indicated the following information.

 (a) The siliceous rocks were hydrothermal genesis with possible terrigenous influences. The Ba content ranged from 115.22 × 10^−6^ to 2572.85 × 10^−6^ (average = 1008.61 × 10^−6^), which was higher than that of typical crustal rocks with average Ba content of 707 × 10^−6^ [77]. The U content ranged from 2.04 × 10^−6^ to 10.91 × 10^−6^ (average = 4.42 × 10^−6^), and was higher than that of typical crustal rocks with average U content of 1.30 × 10^−6^ [77]. These geochemical characteristics agreed with those found in typical hydrothermal sedimentary siliceous rocks [78]. The Ba/Sr ratios ranged from 5.84 to 110.17 (average = 33.34), which agreed with hydrothermal siliceous rocks with Ba/Sr > 1 [79]. The V/(V + Ni) values ranged from 0.10 to 0.90 (average = 0.51), which denoted a reductive environment with V/(V + Ni) > 0.46 [80]. However, the U/Th ratios ranged from 0.22 to 2.91 (average = 0.87), which were slightly lower than those of typical hydrothermal sediments [44]. According to this, the siliceous rocks were shown to be hydrothermal genesis, and this was supported by the plotting in Mn-10 × (Cu + Co + Ni)-Fe discrimination diagram (Figure 10). In addition, the samples deviated from the typical hydrothermal sediments (Figure 10), which possibly indicated the effect of terrigenous materials or volcanic input. So, the siliceous rocks were originated from hydrothermal sedimentation, which were possibly affected by the terrigenous materials or the volcanic input to some extent.

(b) The siliceous rocks deposited in a basin of marginal sea. The siliceous rocks have Sc/Th ratios ranging from 0.55 to 4.33 (average = 1.19), which were consistent with those of siliceous rocks of marginal seas (Sc/Th > 1) [44]. The U/Th ratios ranged from 0.22 to 2.91 with an average of 0.87, and some of them were higher than those of siliceous rock of marginal seas [44]. The Sr/Ba ratios ranged from 0.02 to 0.17 (average = 0.07), which agreed with those of siliceous rocks formed in the basin of a deep ocean or a shallow ocean in retention with Sr/Ba < 1 [82]. According to V/(V + Ni) values averaged 0.51, the sedimentary basin was oxygen deficient, which denoted the Sr/Ba ratios should indicate siliceous rocks of a deep ocean basin. Additionally, the siliceous rocks plotted in Ti-V discrimination diagram (Figure 11(a)) indicated the depositional ocean basin circumstance and tended to be nearer to the basin of a marginal sea in the Ti/V-V/Y discrimination diagram (Figure 11(b)). This meant the siliceous rocks deposited in an ocean basin which was relatively far away from the continent itself. So, the siliceous rock should have been formed in a limited ocean basin in its width, which had also been proposed by the literature [83]. In conclusion, the siliceous rocks were formed in an ocean basin with limited width.

 (3) The rare earth elements of the siliceous rock from Dongxiang ore area were shown in Table 4 and could indicate the following information.

(a) The siliceous rocks were originated from hydrothermal sedimentation, and the sedimentary system was possibly affected by terrigenous input. The ΣREE values ranged from 30.21 × 10^−6^ to 164.16 × 10^−6^ (average = 92.46 × 10^−6^), which agreed with those of typical hydrothermal sedimentary siliceous rocks with ΣREE < 200 × 10^−6^ [85]. Normalized by the PAAS [65], the (La/Yb)_N_ values ranged from 0.10 to 1.47 (average = 0.84), and this was consistent with those of typical hydrothermal sedimentary siliceous rock with (La/Yb)_N_ < 1 [43]. Additionally, some samples with relatively higher (La/Yb)_N_ values possibly indicated terrigenous input during the hydrothermal sedimentation, which was possibly contributed by the continent of magmatic differentiation or crustal contamination [86]. Normalized by PAAS (Figure 12(a)), the REE patterns were shown to be rich in HREE, left-leaning, positive Eu anomaly, and slightly negative Ce anomaly, which agreed with those of hydrothermal sedimentary siliceous rocks. Besides, there were also REE patterns deviated from those of typical hydrothermal sedimentary siliceous rocks partially (Figure 12(b)), which possibly reflected that the sedimentation system had been affected by nonhydrothermal material or volcanic activity. One of them was weakly right-leaning with an obvious positive Eu anomaly, while the other was left-leaning with a weak positive Eu anomaly. So, the hydrothermal sedimentation acted as the predominant mechanism for these siliceous rocks, and the hydrothermal sedimentation process was also affected by terrigenous materials or volcanic inputs.

(b) The siliceous rocks deposited in a width-limited ocean basin of a marginal sea. The ΣREE values had an average of 92.46 × 10^−6^, which was close to that of siliceous rock formed in the marginal sea [43]. Normalized by the PAAS [65], the (La/Yb)_N_ values ranged from 0.10 to 1.47 (average = 0.84), which matched those of siliceous rocks formed both in marginal sea or ocean basins [43]. The *δ*Ce value ranged from 0.92 to 0.98 (average = 0.94), which was consistent with siliceous rock of marginal sea with *δ*Ce from 0.83 to 1.33 [85]. The (La/Ce)_N_ values ranged from 0.98 to 1.09 (average = 1.06), which agreed with those of siliceous rock from a marginal sea [73]. The *δ*Eu values ranged from 1.08 to 2.65 (average = 2.04), which were much higher than those of siliceous rock from the MOR with *δ*Eu being 1.35 [85]. The (La/Lu)_N_ values ranged from 0.09 to 1.26 (average = 0.74), which were consistent with those of siliceous rock from marginal sea with (La/Lu)_N_ values being 0.79 [85]. The siliceous rocks had obvious negative Ce anomaly in the middle ocean ridge [43, 87], which was not apparent in the littoral siliceous rocks. Therefore, the weak negative Ce anomaly in the siliceous rocks denoted that they were possibly formed at the basin of a marginal sea (Figure 12(a)), which was strongly supported by the Al_2_O_3_/(Al_2_O_3_ + Fe_2_O_3_)-(La/Ce)_N_ diagram (Figure 13). So, the siliceous rock deposited in an ocean basin of a marginal sea.

### 4.3. XRD Analysis

**Figure 14:**
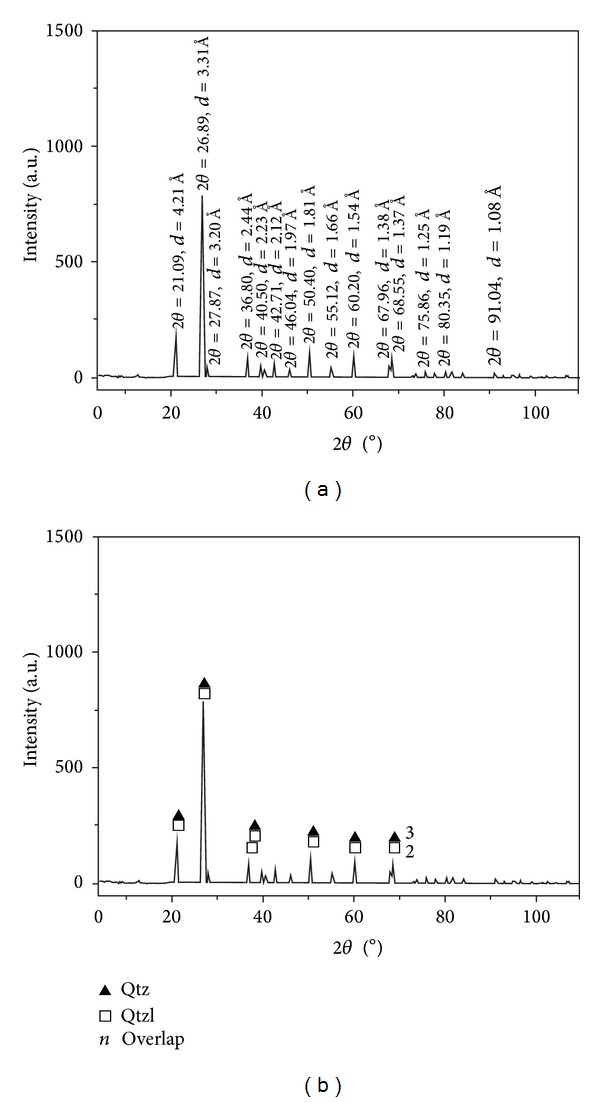
XRD diagram for siliceous rock from Dongxiang ((a) primary analytical result; (b) minerals matched their eight characteristic peaks).

The *α*-quartz were shown to be the major mineral in the X-ray powder diffraction result (Figure 14(a)), which had two kinds of cell parameters (Figure 14(b)). There were trace impurities, such as minerals of carbonate and clay, which were concealed due to their lower concentrations. The first kind of *α*-quartz (Qtz) was hexagonal with space group P3121(152), whose crystal cell parameters were *a* = *b* = 4.913 Å, *c* = 5.405 Å, and *Z* = 3. The second kind of *α*-quartz (Qtzl) was rhombohedral with space group P3221(154), whose crystal cell parameters were *a* = *b* = 4.914 Å, *c* = 5.406 Å, and *Z* = 3. In comparison with standard minerals, the first kind of quartz belonged to standard *α*-quartz with cell parameters*a* = *b* = 4.913 Å, *c* = 5.405 Å, and *Z* = 3, while the crystal cell parameters of the other *α*-quartz (Qtzl) were a little longer than those of the standard *α*-quartz.

The *α*-quartz with lengthening cell parameter was possibly originated from the isomorphous substitution of impurity elements. The crystal cell parameters were similar if they were formed in identical evolution processes with similar circumstances. According to the literature [88, 89], changes in the crystal cell parameters can be contributed by temperature [90], stress [91], transformation into different crystal forms [92], and isomorphous substitution [93]. However, only the isomorphous substitution could lengthen the cell parameter, which would be the most possible mechanism for the *α*-quartz (Qtzl) with lengthening cell parameters. So, the lengthening cell parameters of *α*-quartz possibly came from the isomorphous substitution of impurity elements.

### 4.4. Microarea Analysis

**Figure 15:**
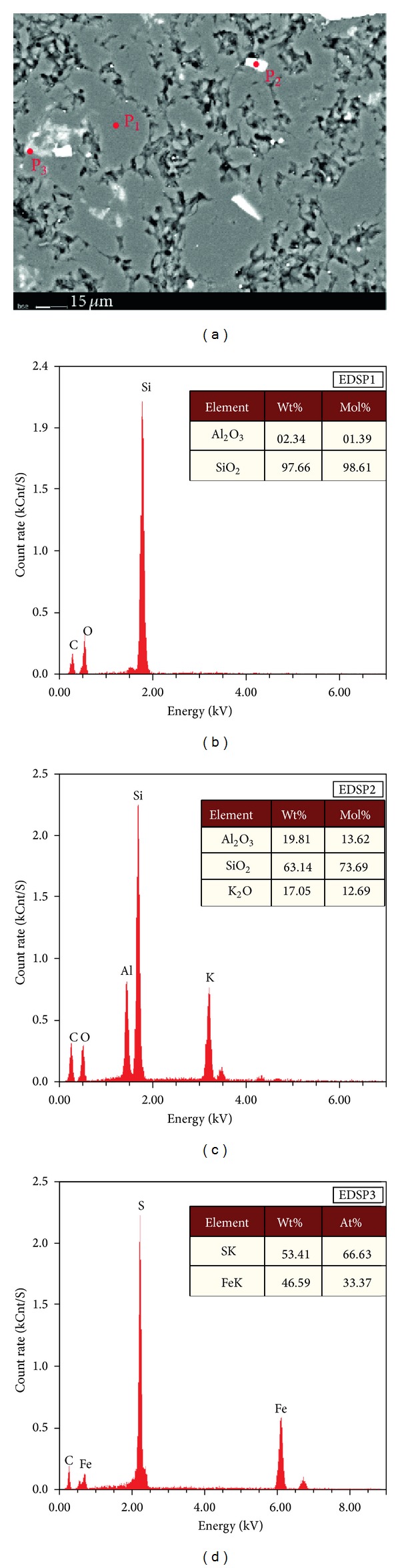
EBSD images and EDS result of the siliceous rock from Dongxiang.

The siliceous rocks were originated from hydrothermal sedimentation. In the EBSD images, the brightness is in positive correlation with the proton number of those elements comprising the minerals [88, 89, 94]. According to this, the low proton number in the SiO_2_ minerals implied low brightness and dark colour, while the clay minerals and metal sulphides had higher brightness with a whiter colour because of their larger proton numbers. In this study, the siliceous rock was mainly composed of silica mineral (Figures 15(a) and 15(b)), a scattering of felsic minerals (Figures 15(a) and 15(c)), and metal sulphides (such as pyrite) (Figures 15(a) and 15(d)). Additionally, the carbon element in the EDS analysis data is brought during the sample production process. The silica mineral (quartz) was low in crystallinity, less automorphic, and fine-grained with typically close-packed texture (Figure 15(a)). This phenomenon matched the hydrothermal sedimentary siliceous rocks which precipitated from the hydrothermal water and crystallised rapidly. Furthermore, there were straight boundaries for the pyrite with scattered distribution (Figures 15(a) and 15(c)), which contributed to evidence of sedimentary genesis as well as the siliceous rocks.

## 5. Discussion

### 5.1. Provenance and Genesis

Siliceous rocks in the Dongxiang area originated from hydrothermal sedimentation, as is strongly supported by both microfabric and geochemical characteristics. The authigenic quartz of the siliceous rocks displays low crystallinity and less automorphism with a close-packed texture (Figures 4(c), 4(d), 15(a), and 15(b)). The thimbleful impurity minerals have a scattered distribution, which originated from syndeposition (Figures 15(a), 15(c), and 15(d)). These microfabric characteristics agree with a hydrothermal genesis, and they also indicate that the primary precipitation rate of silica was too high to crystallise. The siliceous SiO_2_ content ranges from 77.13% to 87.80% (average = 80.41%), which is in agreement with hydrothermal genesis. In addition, their Ba averaged 1008.61 × 10^−6^, U averaged 4.42 × 10^−6^, and ΣREE values averaged 92.46 × 10^−6^. The siliceous rocks originated from hydrothermal sedimentation, as proven by the Fe/Ti ratios that averaged 25.70, the Fe_2_O_3_/FeO ratios that averaged 1.45, the Ba/Sr ratios that averaged 33.34, the V/(V + Ni) value that averaged 0.51, the (La/Yb)_N_ ratios that averaged 0.84, and plotting in the hydrothermal category (Figures 7(a), 7(b), 7(c), 10, and 12). However, there were terrigenous materials or volcanic inputs during the hydrothermal sedimentation, which is supported by the MgO contents averaging 1.14%, Si/Al ratios averaging 9.13, Al/(Al + Fe + Mn) values averaging 0.63, and U/Th ratios averaging 0.87. In addition, the terrigenous materials or volcanic inputs possibly contributed to lengthening cell parameters of the *α*-quartz with isomorphous substitution of impurity elements (Figure 14(b)), which was also indicated by the existence of nonsilica impurities as well as the felsic minerals in the EBSD images (Figures 15(a) and 15(c)). In summary, the microfabric and geochemical characteristics strongly support a hydrothermal genesis for the siliceous rocks in the Dongxiang copper-polymetallic ore area. The siliceous rock arose from hydrothermal sedimentation, and the terrigenous or volcanic materials also contributed to the hydrothermal sedimentation to some extent. Additionally, the siliceous rocks of Qinzhou-Hangzhou joint belt in other published papers [48–50, 59, 95–97] all strongly supported the hydrothermal genesis of siliceous rocks. Therefore, the sedimentary siliceous rocks in the Dongxiang area originated from hydrothermal sedimentation.

There were slight contributions from biological activity during the hydrothermal sedimentation. The hydrothermal activities brought in a large number of the other elements with strong silicon affinity [98] and facilitated biological growth of a large quantity of biological organisms [99]. These organisms carried nonhydrothermal substances because of their lengthy migration and particular elemental needs (e.g., see [100]), and their deaths or metabolites joined in the deposition process with the hydrothermal sediment. This was why there were some samples falling into the biological categories (Figures 7(c) and 9(a)). Although there was biological activity during the hydrothermal sedimentation, the contribution was too weak to affect the majority of the geochemical characteristics. Thus, the siliceous rocks have manifestly hydrothermal genesis with slight biological influences over their geochemical characteristics. 

### 5.2. Formation Environment

The QHJB had a rift trough in the north segment and a subductional ocean basin in the south segment. The siliceous rocks in the Dongxiang copper-polymetallic ore area were formed in a basin of the marginal sea, as strongly supported by its geochemical characteristics, including an Al/(Al + Fe + Mn) values averaging 0.63, MnO/TiO_2_ values averaging 0.92, Al_2_O_3_/(Al_2_O_3_ + Fe_2_O_3_) values averaging 0.82, Sc/Th ratios averaging 1.19, Sr/Ba ratios averaging 0.07, ΣREE values averaging 92.46 × 10^−6^, *δ*Ce values averaging 0.94, *δ*Eu values averaging 2.04, and (La/Lu)_N_ ratios averaging 0.74. In the geochemical discrimination diagrams, the siliceous rocks also fell into the basin of the marginal sea (Figures 8(a), 8(b), 11(b), and 13). The deposition occurred further away from the continent as the U/Th ratios averaged 0.87 and (La/Yb)_N_ ratios averaged 0.84, which suggests an ocean basin as the most likely depositional site. According to the Ti-V and Ti/V-V/Y diagrams (Figures 11(a) and 11(b)), there is also evidence that the siliceous rocks formed in a basin of the marginal sea. Therefore, the siliceous rock in the Dongxiang area was most likely formed, in general, in a basin of the marginal sea. This ocean basin was limited in its width [83], which was possibly a rift trough due to the tensional setting [31]. In this study, the Neopalaeozoic tensional setting was indicated by the large-scale development of Hercynian siliceous rocks (Figure 5), and the terrigenous input, taking part in the sedimentation of Devonian siliceous, to the basin was too narrow to reject the transference of materials from the continent to the basin. Although the siliceous rocks weakly exhibited deposition in the ocean basin category (Figure 11(a)), they were most likely affected by the magmatic activities during the Neopalaeozoic rifting. According to this, there should not be an ocean basin with subduction in the north segment of the QHJB, and the homochronous subduction of ocean basin [35] was possibly only in the south segment. Therefore, the QHJB, separating the Yangtze and Cathaysian plates, was diverse in different segments, which meant a rift trough in the north segment and an ocean basin with subduction in the south segment. 

**Figure 16:**
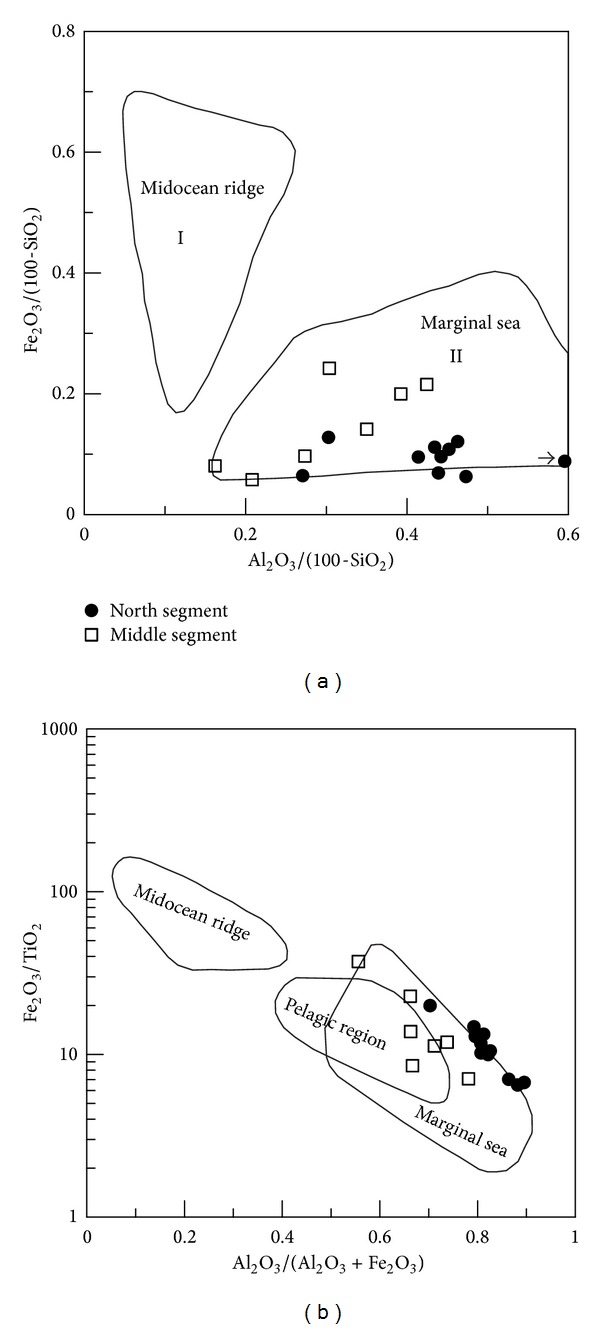
Discrimination diagram of the north and middle QHJB (data of middle segment from [96]; north segment data from this paper).

The ocean basin of the QHJB had a limited width in the north and middle segments. Together with the conjoining of Yangtze and Cathaysian ancient land crusts, the ocean basin of QHJB was closed in the Silurian [33]. Then, the Neodevonian uniform sedimentary veneer covered both the Yangtze and Cathaysian blocks, which is a disconformity in the Silurian strata [34]. Based on the ophiolitic melange in northeast Jiangxi province [28] and the Neopalaeozoic subduction in the south segment [35], there should be a narrow ocean basin that formed during rifting in a NE direction of the QHJB [56]. This ocean basin was composed of the Pingle depression and Qiantang depression in the north segment, which extended to Jiangxi province, south Hunan province, and ultimately the Qin-Fang ocean trough in southeast Guangxi province. Although there was an ocean basin, the width of the basin was too limited to resist the influence of terrigenous clastics. Because of the terrigenous materials, some samples deviated from the category of hydrothermal sedimentary siliceous rocks (Figures 7(a), 10, and 12(b)). According to the discrimination diagrams (Figures 16(a) and 16(b)), the Carboniferous siliceous rocks in the north segment and the Permian siliceous rocks in the middle segment (Figure 2(a)) all fell into the marginal sea category, which indicates that the ocean basin was too limited because it came into being in both the north and middle segments. In addition, the north and middle segments were possibly relatively conformable in width. Hence, the ocean basin of the north and middle segments was limited in its width, which was too narrow to prevent the terrigenous sediment from joining the hydrothermal sedimentation process.

### 5.3. Hydrothermal Metallogenesis

Close relationships were exhibited between the siliceous rocks and the metal ore strata. Many ore deposits are distributed in the joint belt between diverse plates or terranes [101, 102], and the QHJB is *per se* not exceptional [31, 36]. In the QHJB, there are many Neopalaeozoic metal sulphide deposits [103] that were formed in the taphrogenesis depression zone with territorial fracturing as the ore-controlling structure. Many of these ore deposits display close links with siliceous rocks that belong to VMS-type deposits [104], such as the metal sulphide ore deposits in areas of Yongping [105], Dongxiang [48], and Lehua [106]. This indicates the close relationship between metallogenesis and siliceous rocks, especially for the gold ore deposits and copper-polymetallic ore deposits [38]. In the Dongxiang copper-polymetallic ore deposit, the strata of siliceous rocks were distributed at the top of metal sulphide ore bodies or siliceous iron strata (Figure 3(b)), which denotes that the formation of metal sulphide (or siliceous iron) strata is generally followed by the silica strata as a series.

The siliceous rocks and metal sulphides both originated from hydrothermal sedimentation, and their formation had a close relationship with volcanism. During the hydrothermal sedimentation process, the volcanic magma could provide both energy and materials [107, 108]. In this study, the siliceous rocks indicated a tensional setting with siliceous rocks developing at a large scale (Figure 5), and the wide distribution of Neopalaeozoic siliceous rocks denoted a tensional setting in the Hercynian (Figure 6(b)). The tensional setting facilitated magmatic activities that could provide the hydrothermal sedimentation with energy and possible material sources. This is strongly supported by the hydrothermal volcanic contribution of siliceous rocks, which is indicated by the SiO_2_/(K_2_O + Na_2_O) values averaging 35.05, Al_2_O_3_/TiO_2_ ratios averaging 50.03, and the associated geochemical discrimination diagrams (Figures 9(a) and 9(b)). In addition, the K_2_O/Na_2_O and SiO_2_/MgO values deviate from those of typical volcanic siliceous rock, which is possibly because of the contribution of biological factors (Figure 9(a)) and terrigenous inputs. Similar to the geological characteristics of another ore deposit in the nearby Yongping area (Figure 3(a)) [105, 109], these ore deposits had ^206^Pb/^204^Pb ratios ranging from 17.351 to 18.309, ^207^Pb/^206^Pb ratios ranging from 15.416 to 15.661, and ^208^Pb/^204^Pb values ranging from 37.703 to 38.331. Thus, the lead isotope values indicated sources between the upper crust and mantle, and the formation was dated from 330 Ma to 609 Ma. In addition, the *δ* ^34^S values ranged from *‒*10.2‰ to +2.7‰, which agreed with those of the massive sulphide deposits in volcanic thermal springs. As with classic VMS deposits [107, 110, 111], the aforementioned geochemical Yongping characteristics indicated that the ore-forming metal elements came from both volcanic rocks and lower strata, whereas the elemental sulphur came from the convective sea water. Thus, both the siliceous rocks and the metal sulphides arose from hydrothermal sedimentation, and the ore-forming metal elements originated from the lower strata and volcanic rocks. During the convection process, the convective sea water, heated by the lower magmatism, leached the ore-forming metal materials from the lower strata and volcanic rocks. Ultimately, the ore-forming metal materials were precipitated when the hydrothermal water was mixed with the cold seawater with decreasing solubility.

### 5.4. Sedimentary Sequence

The hydrothermal sedimentation process evolved cyclically because of the release of internal energy. The current hydrothermal activities at the ocean bottom were divided into high-temperature black smoker activity (hydrothermal water including mainly massive sulphide) at 400°C to 350°C, medium-temperature white smoker activity (hydrothermal water including silica, barite, and gypsum, among other minerals) at 300 to 100°C, and low-temperature spillway activity (hydrothermal water including pure silica) at below 100°C [112]. The siliceous rocks and metal sulphides were thus formed at different temperatures, which involves a different evolution process with hydrothermal sedimentation at different temperatures. According to the geological sketch histogram (Figure 3(b)), the siliceous rocks were present in alternating layers, as were wall rocks and metal sulphide (or siliceous iron) strata, and the strata of siliceous rocks overlaid conformably the massive sulphide or siliceous iron formation all the time. At the bottom of the sulphide strata, there were conformable tuff strata that overlaid the marine volcanic rocks. As a whole, there were several sedimentary cycles in the Dongxiang ore deposit, and each of the cycles possibly involved marine volcanic rocks, volcanic tuff, metal sulphide, and siliceous rocks from the bottom to the top. Thus, the hydrothermal sedimentary strata, as well as the other sedimentary rocks, display periodic sedimentation in the Dongxiang area, including siliceous rocks and massive metal sulphides.

Sedimentary cycles act as an indication of interior dynamic geological evolution. From the bottom to the top, each sedimentary cycle included marine volcanic rock, volcanic tuff, ore strata (including massive sulphide or siliceous iron), and siliceous rock. The normal marine sedimentary rocks (e.g., limestone, mudstone, or clastic rocks) were formed at the end of the continuous sedimentary sequence, which was related to the interior dynamic geological evolution. Therefore, the sedimentary sequence in the Dongxiang area could be divided into several cycles that represent the release of energy from the inner earth. In every normal sedimentary cycle, the internal energy was released rapidly at first with high intensity and then slowed down gradually as the cycle continued. The initial energy released was stronger with the volcanic eruption and associated ash, and this contributed to the development of volcanic rocks followed by the volcanic breccias or tuff at the ocean bottom. After the volcanic eruption, there was high-temperature hydrothermal water with the metal sulphides as the main precipitation. The ore strata were formed at this time. When the release of energy from the inner earth became less violent, the hydrothermal water changed to become silica-rich and lacking in metal sulphides with a relatively lower temperature. In addition, the sedimentary sequences for the hydrothermal activities should be distinct if they arose from different geological settings. If the volcanism was too weak to produce volcanic ash or stagnates and was suddenly without volcanic ash, the volcanic tuff layer would disappear with a sedimentary sequence, including marine volcanic rock, massive sulphides, and siliceous rocks. In another scenario, if the energy coming from interior magmatism was too weak to ensure a high-temperature sulphide smoker immediately after the volcanic eruption, there might be a sedimentary sequence with marine rocks, volcanic tuff, and siliceous rocks from the bottom to the top. In conclusion, the marine sedimentary rocks, including those marine volcanic rocks, volcanic tuff, metal sulphides, and siliceous rocks, were contributed by the release of internal energy from the earth, and their combined sequences represent the cyclic release of internal energy.

## 6. Conclusions

(1) The siliceous rocks originated from hydrothermal sedimentation. The siliceous rocks had SiO_2_ contents ranging from 77.13% to 87.80% with an average of 80.41% and exhibited hydrothermal genesis for their quartz grains with low crystallinity, less automorphism, and a close-packed texture. Identifiably hydrothermal characteristics were also observed in the geochemical composition, such as the Ba averaging 1008.61 × 10^−6^, U averaging 4.42 × 10^−6^, ΣREE values averaging 92.46 × 10^−6^, Fe/Ti ratios averaging 25.70, Al/(Al + Fe + Mn) values averaging 0.63, (Fe + Mn)/Ti values averaging 26.89, Fe_2_O_3_/FeO ratios averaging 1.45, Ba/Sr ratios averaging 33.34, and (La/Yb)_N_ values averaging 0.84.

(2) The Neopalaeozoic Qinzhou-Hangzhou joint belt was diverse in different segments, and the siliceous rocks of the north Qinzhou-Hangzhou joint belt were deposited in a basin of the marginal sea. The siliceous rocks were formed in the marginal sea, which is indicated by MnO/TiO_2_ ratios averaging 0.92, Al/(Al + Fe) values averaging 0.64, Sc/Th ratios averaging 1.19, U/Th ratios averaging 0.87, and *δ*Ce values averaging 0.94. In addition, the ocean basin of the north and middle segments of Qinzhou-Hangzhou joint belt was limited in its width without subduction and accordingly could not resist terrigenous input during the hydrothermal sedimentation. 

(3) The siliceous rock exhibited close relationships with volcanic activity, which could provide energy and material sources. The hydrothermal sedimentation of the siliceous rocks was influenced by volcanic activity, and this was indicated by SiO_2_/(K_2_O + Na_2_O) values averaging 35.05 and Al_2_O_3_/TiO_2_ values averaging 50.03. The sedimentary rocks of the Dongxiang copper-polymetallic ore deposit are the product of the release of internal earth energy, and their alternative existence represents cyclical earth energy release.

(4) There are two types of cell parameters for the *α*-quartz grains. In the siliceous rocks, the majority mineral was *α*-quartz with two types of crystal cell parameters. The first type of *α*-quartz had Pdf card number 75-0443 and space group P3121(152) with agreement to standard *α*-quartz, whereas the other type of *α*-quartz had Pdf card number 65-0466 and space group P3221(154) with slightly elongated cell parameters.

## Figures and Tables

**Table 1:** Localities of siliceous rocks in Qin-Hang joint belt.

	Guangdong province	Guangxi province	Hunan province	Jiangxi province	Zhejiang province
Mesopaleozoic		Longsheng	Taoyuan	Wuning	

Neopaleozoic	Lechang, Xinyi, LianJiang, Huazhou, Xingning, Meixian, Jiaoling, Yangshan, Huaiji, and Guangning	Sanjiang, Hexian, Luocheng, and Longsheng	Shimen, Lingling, Chaling, Shimen, Anhua, Xupu, Guiyang Zhengyuanling, Guiyang Dajiangbian, Guiyang Sizhoushan, and Guiyang Tianzidi	Wuning, Guangfeng A, Yiyang Qigong, Xiushui, North Xiushui, Wuning, Pengze, Boyang, Dexing, Yiyang, Pingxiang, Guangfeng B, Xiajiang, and Taiheqiao	Fuyang Zhangcun, Fuangyang Luojiamen, Fuyang Hongchicun, Longyou, Changshan, Zhuji, Fuyang Zhongjiazhuang, and Chunan

Cambrian	Lechang	Jiuxiu, East Hexian, Hexian Tanchong, Rongxian, West Damingshan, Zhongshan, Liuzhou, Rongshui, Luocheng, Quanzhou, and Yongfu	Shimen, Luxi, Anhua Yanxi, Anhua Tanxi, Yuanling, Shuangfeng, Jianghua, and Rucheng	Xiushui, Yushan, Shangrao, Wuning, Congyi, and Xingguo	Jiangshan A, Anji, Jiangshan B, and Yuhang

Ordovician	Taishan, Dongyuan, and Qujianoug	Quanzhou, Gongcheng	Taojiang, Shuangfeng, Anhua, Dongan Xiejiawan, Dongan Sushuichong, Qidong Shuangjiakou, and Qidong Shimenkou	Guangfeng, Congyi, Ninggang, Yongxin Hangjianglongxi, Yongxin Shikou, and Yongxin Pulong	Kaihua, Longyou, Linan Shangluojia, Linan Yakou, and Chunan Tantou

Silurian		Fangcheng	Anhua		

Devonian	Yunfu Shimen, Yunfu Dajiangping, Luoding Xinrong, Huaiji, Lianxian, Lechang, and Renhua	Nandan, Xiangzhou, Wuxuan, Nanning, Wuming, Shangling, Ningming, Longzhou, Daxin, Jingxi, Debao, Tianlin, Nonglin, Hengxian, Longzhou, Napo, Hexian, Qinzhou, and Beiliu	Shaodong, Qiyang		

Carboniferous	Longchuan, Haifeng, Meixian, Wuhua, Zijin, Lianxian Dashiyan, Dongfang, LianXian Zhoushui, Lianping, Lianxian Outang, Yangshan, Ruyuan, Heping, Huiyang, Pingyuan, Lianxian Waidong, Jiaoling, Lianxian Panhai, and Yingde	Liucheng Anlecun, Luocheng, Liucheng Taiping, Huanjang, Luocheng, Xingan, Quanzhou, Yishan, Nandan, Luzhai, Jingxi, Pingguo, Xilin, Nonglin, Tianlin, Tiandong, and Tiandeng	Lianyuan	Pingxiang, Anfu, Yongxin, Shuichuan, Gaoan, and Dongxiang	

Perminian	Lianxian, Yangshan, Qujiang, Yangchun Chunwan, Yangchun Longyungang, Renhua, Longmen, Enping, and Menxian	Qinzhou, Shanglin, Donglan, Pingle, Bingyang, Hechi, Yishan A, Liucheng, Duan, Heshan, Shanglin, Yishan B, Hengxian, Shangsi, and Congzuo	Shangzhi, Chenxi Zhonghuopu, Chenxi Wulidun, Lianyuan, Shaoyang, Leiyang, and Shaodong	Xiushui, Ruichang, Lianshan, and Qianshan	Tonglu, Changxing

Triassic		Pingguo, Tianlin, and Napo	Zhongfang	Wanzai	

Jurassic	Huaiji				Songyang, Lishui

Cretaceous			Hengyang		

*Note,* Data were calculated from the literature [60–64]; the siliceous rocks were calculated with formation and county as the temporal unit and spatial unit, and the siliceous rocks located in one place with diverse Formations will be differentiated with capitalization like “A, B, C, etc.” or addition of subplace name.

**Table 2:** Major element analysis data for siliceous rocks from Dongxiang area (%).

No.	SiO_2_	TiO_2_	Al_2_O_3_	Fe_2_O_3_	FeO	MnO	MgO	CaO	Na_2_O	K_2_O	P_2_O_5_	LOI	Total
DX013a	79.10	0.15	8.66	2.00	1.76	0.11	2.50	0.50	1.30	1.87	0.07	1.79	99.81
DX013b	80.35	0.17	8.54	2.20	1.75	0.12	1.42	0.53	1.32	1.65	0.08	1.65	99.78
DX015	79.49	0.20	9.71	1.30	1.56	0.05	0.75	0.39	3.00	1.89	0.07	1.35	99.76
DX016	77.13	0.22	10.12	2.20	2.06	0.10	1.15	0.58	3.52	0.82	0.08	2.06	100.04
DX020	79.44	0.19	9.30	2.23	1.70	0.10	1.72	0.53	0.93	1.66	0.09	1.94	99.82
DX024	79.67	0.20	8.93	1.41	2.15	0.09	0.73	0.51	1.10	2.78	0.08	2.46	100.11
DX025	79.48	0.13	5.56	1.33	2.10	0.32	1.27	2.48	0.65	1.25	0.07	5.16	99.80
DX026	82.84	0.11	5.20	2.20	1.58	0.27	0.76	1.27	0.49	0.48	0.07	4.60	99.87
DX027	78.82	0.23	13.26	1.55	0.52	0.01	0.51	0.38	0.66	1.54	0.07	2.19	99.73
DX028	87.80	0.10	5.65	1.48	0.46	0.12	0.60	0.54	0.71	1.04	0.07	1.27	99.84

Aver.	80.41	0.17	8.49	1.79	1.56	0.13	1.14	0.77	1.37	1.50	0.07	2.45	99.86

**Table 3:** Trace element analysis result (×10^−6^) and related geochemical indices for siliceous rocks from the Dongxiang area.

No.	Li	Be	Sc	V	Cr	Co	Ni	Cu	Zn	Ga	Rb	Sr	Y	Zr	Nb	Mo	Cs	Ba	Hf	Ta	Pb	Th	U
DX013a	37.09	0.91	6.03	40.90	125.63	10.25	19.67	18.86	206.87	8.55	106.07	34.82	13.28	182.02	10.74	0.61	4.32	1372.82	4.77	0.79	252.22	8.83	2.31
DX013b	46.89	0.84	5.11	33.74	194.22	9.42	17.04	24.13	1034.24	7.41	95.08	31.45	11.66	155.86	9.29	0.59	3.42	1220.98	4.12	0.68	2695.17	7.58	2.04
DX015	32.38	1.02	6.96	43.68	46.98	55.37	105.27	22.82	103.87	8.71	99.71	115.82	11.73	158.47	11.33	0.60	4.18	1177.75	4.28	2.44	465.90	9.40	2.85
DX016	33.56	0.90	6.54	38.85	223.83	10.91	22.79	80.26	234.92	9.53	47.81	58.70	16.17	206.38	12.33	0.83	1.97	560.73	5.44	0.90	766.46	10.39	2.46
DX020	48.74	1.13	5.70	35.76	32.18	50.47	92.84	339.76	856.62	9.34	110.09	25.90	14.46	182.84	9.90	1.00	5.07	1612.74	4.85	2.10	731.93	10.35	2.33
DX024	68.84	2.13	6.25	32.86	66.59	7.72	3.92	8.83	122.67	9.20	149.31	23.35	17.59	216.66	7.48	4.89	12.55	2572.85	4.82	0.52	55.51	6.34	3.56
DX025	113.30	0.99	5.96	29.12	3.90	50.15	87.32	7.17	281.43	6.24	77.36	32.56	16.34	95.81	3.52	1.82	5.54	598.91	2.14	1.63	91.82	3.75	10.91
DX026	202.99	0.77	9.45	31.27	164.82	3.14	3.54	12.18	621.12	4.99	41.99	19.46	24.63	7.67	0.31	0.24	3.64	115.22	0.20	0.03	207.75	2.18	2.77
DX027	65.69	1.84	5.22	33.81	5.52	35.08	54.01	71.78	30936.72	13.04	107.55	23.08	22.70	207.50	7.54	3.81	11.87	134.78	4.63	1.29	3129.25	7.29	7.60
DX028	107.51	1.08	4.31	18.75	3.83	101.80	171.69	37.44	344.71	5.09	66.78	21.91	14.19	107.26	3.44	2.85	4.72	719.29	2.29	2.62	169.37	4.47	7.36

Aver.	75.70	1.16	6.15	33.87	86.75	33.43	57.81	62.32	3474.32	8.21	90.17	38.71	16.27	152.05	7.59	1.72	5.73	1008.61	3.76	1.30	856.54	7.06	4.42

**Table 4:** REE analysis results (×10^−6^) and related geochemical indices for siliceous rocks from the Dongxiang area.

No.	La	Ce	Pr	Nd	Sm	Eu	Gd	Tb	Dy	Ho	Er	Tm	Yb	Lu	ΣREE	(La/Ce)_N_	(La/Yb)_N_	*δ*Ce	*δ*Eu	(La/Lu)_N_
DX013a	22.90	44.95	5.31	19.78	3.61	0.95	2.88	0.45	2.44	0.51	1.50	0.25	1.56	0.27	107.35	1.06	1.08	0.94	1.39	0.97
DX013b	22.55	42.95	5.01	18.57	3.41	1.08	2.59	0.39	2.10	0.45	1.26	0.20	1.26	0.23	102.05	1.09	1.32	0.93	1.72	1.11
DX015	21.03	42.05	4.90	17.92	3.27	1.06	2.54	0.39	2.17	0.44	1.31	0.22	1.46	0.24	98.99	1.04	1.06	0.96	1.73	1.01
DX016	35.23	72.14	8.15	29.98	5.13	1.34	3.89	0.57	3.04	0.61	1.71	0.28	1.77	0.32	164.16	1.02	1.47	0.98	1.41	1.26
DX020	19.38	36.98	4.28	16.00	3.00	0.66	2.71	0.44	2.53	0.54	1.59	0.27	1.74	0.30	90.41	1.09	0.82	0.94	1.08	0.74
DX024	19.45	35.78	4.14	16.22	3.32	2.01	3.11	0.52	2.91	0.62	1.84	0.29	1.81	0.32	92.35	1.13	0.79	0.92	2.94	0.69
DX025	10.55	21.51	2.72	11.05	2.52	1.49	2.78	0.47	2.78	0.56	1.63	0.26	1.71	0.31	60.32	1.02	0.46	0.93	2.65	0.39
DX026	2.99	6.37	0.86	3.95	1.36	1.00	2.53	0.60	4.07	0.90	2.52	0.38	2.28	0.38	30.21	0.98	0.1	0.92	2.54	0.09
DX027	23.36	45.28	5.44	21.73	4.67	2.39	4.92	0.75	3.99	0.77	2.17	0.34	2.19	0.37	118.37	1.08	0.79	0.93	2.35	0.72
DX028	11.46	22.51	2.74	11.08	2.30	1.27	2.31	0.40	2.34	0.50	1.41	0.24	1.58	0.29	60.42	1.06	0.53	0.93	2.6	0.45

Aver.	18.89	37.05	4.35	16.63	3.26	1.32	3.02	0.50	2.84	0.59	1.69	0.27	1.74	0.30	92.46	1.06	0.84	0.94	2.04	0.74

N represent normalized by PAAS [65]; *δ*Ce and *δ*Ce come from *δ*Ce = Ce/Ce* = Ce_N_/(La_N_ × Pr_N_)^1/2^ and *δ*Eu = Eu/Eu* = Eu_N_/(Sm_N_ × Gd_N_)^1/2^ [66].
